# Supplementary material for: Iterative improvement in the automatic modular design of robot swarms
Source: PeerJ Comput Sci. 2020 Dec 7;6:e322. doi: 10.7717/peerj-cs.322 (PMC7924708; doi:10.7717/peerj-cs.322)
Supplement: Supplemental Information 3 [file peerj-cs-06-322-s003.zip › argos3/doc/api/standalone/a00602_source.html]

ARGoS: plugins/simulator/physics\_engines/dynamics2d/dynamics2d\_cylinder\_model.cpp Source File


- Main Page
- Related Pages
- Namespaces
- Classes
- Files

- File List
- File Members

# plugins/simulator/physics\_engines/dynamics2d/dynamics2d\_cylinder\_model.cpp

Go to the documentation of this file.

```
00001 
00007 #include "dynamics2d_cylinder_model.h"
00008 #include "dynamics2d_gripping.h"
00009 #include "dynamics2d_engine.h"
00010 
00011 namespace argos {
00012 
00013    /****************************************/
00014    /****************************************/
00015 
00016    CDynamics2DCylinderModel::CDynamics2DCylinderModel(CDynamics2DEngine& c_engine,
00017                                                       CCylinderEntity& c_entity) :
00018       CDynamics2DStretchableObjectModel(c_engine, c_entity) {
00019       /* Get the radius of the entity */
00020       Real fRadius = c_entity.GetRadius();
00021       /* Create a circle object in the physics space */
00022       const CVector3& cPosition = GetEmbodiedEntity().GetOriginAnchor().Position;
00023       CRadians cXAngle, cYAngle, cZAngle;
00024       GetEmbodiedEntity().GetOriginAnchor().Orientation.ToEulerAngles(cZAngle, cYAngle, cXAngle);
00025       /*
00026        * Create body and shapes
00027        */
00028       cpBody* ptBody;
00029       if(GetEmbodiedEntity().IsMovable()) {
00030          /* The cylinder is movable */
00031          SetMass(c_entity.GetMass());
00032          /* Create the body */
00033          ptBody = cpSpaceAddBody(GetDynamics2DEngine().GetPhysicsSpace(),
00034                                  cpBodyNew(GetMass(),
00035                                            cpMomentForCircle(GetMass(),
00036                                                              0,
00037                                                              fRadius + fRadius,
00038                                                              cpvzero)));
00039          ptBody->p = cpv(cPosition.GetX(), cPosition.GetY());
00040          cpBodySetAngle(ptBody, cZAngle.GetValue());
00041          /* Create the shape */
00042          cpShape* ptShape = cpSpaceAddShape(GetDynamics2DEngine().GetPhysicsSpace(),
00043                                             cpCircleShapeNew(ptBody, fRadius, cpvzero));
00044          ptShape->e = 0.0; // no elasticity
00045          ptShape->u = 0.7; // lots contact friction to help pushing
00046          /* The shape is grippable */
00047          SetGrippable(new CDynamics2DGrippable(GetEmbodiedEntity(),
00048                                                ptShape));
00049          /* Set the body so that the default methods work as expected */
00050          SetBody(ptBody, c_entity.GetHeight());
00051          /* Friction with ground */
00052          SetLinearFriction(0.0f, 1.49f);
00053          SetAngularFriction(0.0f, 1.49f);
00054       }
00055       else {
00056          /* The cylinder is not movable */
00057          /* Create a static body */
00058          ptBody = cpBodyNewStatic();
00059          ptBody->p = cpv(cPosition.GetX(), cPosition.GetY());
00060          cpBodySetAngle(ptBody, cZAngle.GetValue());
00061          /* Create the shape */
00062          cpShape* ptShape = cpSpaceAddShape(
00063             GetDynamics2DEngine().GetPhysicsSpace(),
00064             cpCircleShapeNew(ptBody,
00065                              fRadius,
00066                              cpvzero));
00067          ptShape->e = 0.0; // No elasticity
00068          ptShape->u = 0.1; // Little contact friction to help sliding away
00069          /* This shape is normal (not grippable, not gripper) */
00070          ptShape->collision_type = CDynamics2DEngine::SHAPE_NORMAL;
00071          /* Set the body so that the default methods work as expected */
00072          SetBody(ptBody, c_entity.GetHeight());
00073       }
00074    }
00075 
00076    /****************************************/
00077    /****************************************/
00078 
00079    REGISTER_STANDARD_DYNAMICS2D_OPERATIONS_ON_ENTITY(CCylinderEntity, CDynamics2DCylinderModel);
00080 
00081    /****************************************/
00082    /****************************************/
00083 
00084 }
```

---

Generated on 10 Jul 2018 for ARGoS by 
 1.6.1
